# Lectures on Diseases of the Chest

**Published:** 1845-04

**Authors:** J. R. Buck

**Affiliations:** Lecturer on the Theory and Practice of Medicine in the Louisville Summer School of Medicine


### WESTERN JOURNAL

OF

### MEDICINE AND SURGERY.

APRIL, 1845.

Art. I.—*Lectures on Diseases of the Chest.* By J. R. Buck, M.D., Lecturer on the Theory and Practice of Medicine in the Louisville Summer School of Medicine.

### LECTURE 1st.

The organs of the chest can only be examined through the walls of this cavity; and to be able to do this with any degree of accuracy, it is absolutely essential that their healthy relations be understood. To facilitate this understanding it is customary to map out upon the chest a number of artificial regions by imaginary lines drawn from point to point on its circumference. There are four of these regions anteriorly—four posteriorly, and three laterally upon each side of the median line.

*Anteriorly.*—1st. Clavicular region—space covered by clavicle; the post-clavicular depression is also embraced in this region.

Infra-clavicular region, extending from clavicle to fourth rib.

Mammary region, from fourth to eighth rib.

Infra-mammary region, from eighth rib to base of chest.

*Posteriorly.*—Acromial, from spine of scapula to the upper margin of post-clavicular region in front.

Scapular region, space covered by the scapula.

Inter-scapular region, between the bone and spinous processes of the vertebræ.

Infra-scapular region, from lower edge of this bone to base of chest.

*Laterally.*—Axillary region, arm-pit to fourth rib.

Middle lateral region, from fourth to eighth rib.

Inferior lateral, from eighth rib to base of chest.

Thus it will be seen that there are twenty-two regions, eleven on each side of the median line.

Having ascertained the localities of the different regions, the next objects of study are their contents, what organ, organs or parts of organs are contained in them, the relation of the individual parts to different portions of the regions; and 2dly, the evidences of a healthy condition of these organs. In the clavicular regions you have only the apices of the lungs; conical projections occupying the small space between the anterior and posterior walls of the chest. These regions derive their chief importance from being the point where tuberculous deposit almost uniformly first occurs. In the infra-clavicular region the upper lobes of the lungs, the larger bronchi, the base of the heart, and the ascending aorta and pulmonary artery, are found, the base of the heart, bronchi, and bloodvessels existing near and under the sternum. It is important to mark the contents of these regions; first, that in the diagnosis of cardiac diseases, more especially diseases of the valves, it is essential that you have an accurate knowledge of the relation of each individual portion of the heart as well as of the ascending aorta and pulmonary artery to the parietes of the chest. Secondly, The larger bronchi existing here, the respiratory sounds differ somewhat from those heard in the superior regions. And lastly the upper lobes of the lungs, although the same in structure as that in the clavicular region, being so much thicker in consequence of the gradual expansion of the chest from above downwards, modifies somewhat the physical signs either of a healthy or morbid condition.

In the mammary region, the middle lobes of the lungs and heart, are situated; the heart being principally in the left mammary region and beneath the sternum.

In the infra-mammary regions, the lower lobe of the lungs, and portion of liver on right side, and stomach and spleen on left side, exist.

Posteriorly—Acromial region. This region like the clavicular is occupied by the apices of the lungs—but the chest at this point is covered by dense muscle—hence in percussing the chest in the two regions, a different degree of resonance is elicited.

*Scapular region.* Here the middle and upper lobes of the lungs are both found—but an examination posteriorly has to be made through the scapula. Inter-scapular region.—Roots of lungs and large bronchi—the bronchi being nearer the surface *here* than anteriorly, the blowing sound of respiration as well as the morbid sounds formed in the larger bronchial tubes, are heard most distinctly in this region.

*Infra-scapular region.* Lower lobes of lungs– liver on right, and stomach and spleen on left,—the precise point upon the chest at which the lungs and liver come in contact varies so much in different subjects, and in the same subject at different periods of the respiratory process, that we are compelled to resort to percussion to determine it in each individual case.

*Laterally.—Axillary region.* Upper lobes of lungs. Middle lateral—middle lobes of lungs.

Inferior. Lateral—lower lobes of lungs and liver on right side; the stomach and spleen on the left.

In the healthy chest, such are the contents of the various regions. But in certain diseases, both of the abdominal and thoracic viscera, these relations are very materially changed. The physiological or healthy condition of the contents of the thoracic regions can only be ascertained certainly by physical examination; for although the general symptoms when present indicate disease, their absence is by no means proof conclusive of the absence of disease. For here, as elsewhere, disease may exist in a state of latency in regard to the general symptoms, and it is only by the conformation of the chest, and by the sounds elicited upon auscultation and percussion, that we are enabled to determine the healthy condition of these organs.

Conformation of the Chest.—The healthy chest is an irregular truncated cone, the apex being above and the base below: on its external surface exist various irregularities, dense muscles, scapula, clavicle, and in the female the mammary glands. Anteriorly there is a slight projection at the apex, caused by the clavicle, with a depression above and behind it: this depression varies with the degree of muscular and adipose development in the subject; immediately beneath the clavicle from that bone to fourth rib, a slight depression or concavity exists—from fourth rib to base of chest is a regular convexity: a slight prominence exists in the precordial region, where the heart is in contact with the ribs; the chest is widest, at the lower margin of the seventh rib, the pressure of the clothes slightly contracting it from this to the base.

*Laterally*—The chest is regularly convex from the axilla to the base.

*Posteriorly*—The irregularities are so great that they can only be correctly studied upon the subject; the scapula is the most projecting point upon this surface—all the other regions are compressed when compared with this—the inter-scapular most, the infra-scapular next, and the acromial least. During expiration and inspiration the chest dilates and contracts freely—the ribs rising in inspiration and falling in expiration.

*Percussion.*—The healthy chest when struck, yields a clear resonant sound, in those regions occupied by the lungs. This resonance is owing to the presence of atmospheric air in the bronchial tubes and air-cells of the lungs.

*Clavicular.*—The resonance, though perfect in these regions, is not so intense as in the inferior regions for reasons already mentioned.

*Infra-clavicular.*—Resonant throughout, save at the centre of the inferior portions over the base of the heart, where there is a dull sound on percussion.

*Mammary regions.*—Intense resonance over the pulmonary structure. But under the sternum, three-fourths of an inch to its right, and extending to the left a little beyond the cartilaginous extremities of ribs, and as low down as the upper margin of 6th rib—the limits of the heart, the sound upon percussion is dull. The heart is partially overlapped by the thin margin of the lungs, and although when forcibly percussed yielding a dull sound, it is not the perfect dullness elicited from that part of the organ in immediate contact with the parietes of the chest.

*Infra-mammary regions.*—That portion of these regions containing the lower lobes of lungs yields a clear resonant sound on percussion. Over the liver on the right side, the sound is dull, and in the lower part of the left side, there is an irregular dullness depending on the condition of the stomach and spleen.

*Laterally.*—In the two upper regions the resonance is loud and perfect; in the inferior lateral, clear in the upper portion, dull over the liver, and irregularly dull at the corresponding point upon the left side, as may be the condition of the stomach and spleen.

*Posteriorly.*—Resonance is not as good at any point as in the anterior and lateral regions, the dense muscles and bones being obstacles to its transmission from the organs of the chest to the ear. Still the same character of sounds is preserved—only diminished in intensity. It is required to percuss with more force in these regions, that the natural sound of the organs may be transmitted through the thick walls of the chest. Percussing lightly, the dull sounds of the bones and muscles is alone elicited. The lungs being at all points in contact with the posterior walls of the chest, a resonant sound is heard throughout, only modified by the irregularities on its surface.

*Auscultation.*—An accurate knowledge of the anatomy of the lungs is essential to the student before he is even prepared to enter upon the study of auscultation. Almost the entire bulk of the healthy lungs is formed by air. The bronchial tubes ramify through the lungs, and terminate in expanded pouches or air cells, called vesicles. In the large or primitive bronchi, the cartilaginous rings are dense and very close together—the ligamentous tissue connecting these rings is also dense—but as the tubes ramify they become smaller, and the distance between them increases, until in the minute extremities and cells, the muscular and mucous tissues only remain. The friction of the air against the sides of these tubes in inspiration and expiration, produces certain sounds called the respiratory sounds. The sound produced in the larger bronchi, composed of dense cartilage, ligament, &c., differs from that found in the extremities and cells composed only of mucous and muscular tissue. This has given rise to a division of the respiratory sounds into the bronchial, or blowing sound, and the vesicular sound. The first being produced in the large tubes, while the last is produced in the minute extremities, and as its name implies in the vesicles of the lungs. During inspiration both these sounds are heard, the external pressure forcing the air with great suddenness into the extreme cells. In expiration only the blowing sound is heard—the air being forced out of the vesicles so slowly by their gradual contraction, that the friction is not sufficient to produce any appreciable sound. The bronchial or blowing sound of inspiration resembles somewhat that made by contracting the mouth and inhaling air with some little force; it is heard most distinctly about the middle of either interscapular region, or anteriorly, about the lower part of the centre of the infra-clavicular region. The vesicular sound has been compared to the gentle murmur of a distant breeze

—hence it is frequently called vesicular murmur. This is heard at every point of a healthy chest, but most distinctly in the anterior lower and lateral regions. The presence of this sound is conclusive as to the healthy condition of the substance of the lungs. The blowing sound of expiration is a shorter, more abrupt, and feebler sound than that of inspiration; in many healthy subjects when breathing naturally and easily this sound is not heard at all. But in others it is heard with great distinctness, and in the first class it can easily be heard by slightly accelerating the respiration.

As above mentioned, the presence of the vesicular murmur indicates unerringly a healthy condition of the parenchyma of the lung, while the bronchial or blowing sound is altered in character or intensity, by the slightest disease of the larger tubes. The healthy respiratory sounds differ in different ages —thus in infancy it is so much shriller and louder as to have obtained a distinct appellation, peurile respiration; and this variety occurring in an adult is an evidence of disease—so that what is a physiological condition at one age, becomes a pathological condition at another. When an individual whose lungs are healthy speaks, the voice is heard with some distinctness over the large bronchi, and a diffused roaring over the vesicular portion of the chest. The presence of these different sounds, as they have been described, is the evidence upon auscultation of a healthy condition of the contents of the different regions. The heart I do not now mention, as it will be the subject of distinct consideration hereafter.

